# A comprehensive analysis of PANoptosome to prognosis and immunotherapy response in pan-cancer

**DOI:** 10.1038/s41598-023-30934-z

**Published:** 2023-03-08

**Authors:** Lingling Zhuang, Qiran Sun, Shenglan Huang, Lanyan Hu, Qi Chen

**Affiliations:** 1 Department of Obstetrics and Gynecology, Second Affiliated Hospital of Nanchang University, No. 1 Minde Road, Nanchang, 330006 Jiangxi People’s Republic of China; 2 Department of Obstetrics and Gynecology, The First Affiliated Hospital of Nanchang University, No. 17 Yongwaizheng Street, Nanchang, 330006 Jiangxi People’s Republic of China; 3 Department of Oncology, Second Affiliated Hospital of Nanchang University, No. 1 Minde Road, Nanchang, 330006 Jiangxi People’s Republic of China

**Keywords:** Cancer genomics, Immunotherapy

## Abstract

PANoptosis, a programmed cell death, shares key characteristics of apoptosis, pyroptosis, and necroptosis. Accumulating evidence suggests that PANoptosis plays a crucial role in tumorigenesis. However, the respective regulation mechanisms in cancer are so far unclear. Using various bioinformatic approaches, we comprehensively analyzed the expression patterns, genetic alterations, prognostic value, and immunological role of PANoptosis genes in pan-cancer. Expression of the PANoptosis gene, PYCARD, was validated based on the Human Protein Atlas database and real-time quantitative reverse transcription polymerase chain reaction (RT-PCR). We found that PANoptosis genes were aberrantly expressed in most cancer types, which was consistent with the validation of PYCARD expression. Concurrently, PANoptosis genes and PANoptosis scores were significantly associated with patient survival in 21 and 14 cancer types, respectively. Pathway analysis showed that PANoptosis score was positively correlated with pathways linked to immune and inflammatory responses in pan-cancer, such as IL6-JAK-STAT3 signaling, the interferon-gamma response, and IL2-STAT5 signaling. In addition, the PANoptosis score was significantly correlated with the tumor microenvironment, the infiltration levels of most immune cells (i.e.NK cells, CD8+ T cells, CD4+ T cells, DC cells), and immune-related genes. Furthermore, it was a predictive indicator of immunotherapy response in patients with tumors. These insights substantially improve our understanding of PANoptosis components in cancers and may inspire the discovery of novel prognostic and immunotherapy response biomarkers.

## Introduction

Programmed cell death (PCD) is an evolutionarily conserved process that plays a critical role in organism development and tissue homeostasis^1^. PCD by apoptosis was suggested to act as a natural barrier against cancer progression; however, most tumor cells can develop resistance to apoptosis and thus to therapy^2,3^. Pyroptosis and necroptosis are two additional well-researched PCDs. Pyroptosis is mediated by the gasdermin family which depends on the inflammasome^4^, whereas necroptosis is mediated by receptor-interacting serine/threonine-protein kinase 1 (RIPK1) and RIPK3 as well as the downstream effector of the mixed lineage kinase domain-like pseudokinase^5^. PANoptosis, which is controlled by a complex termed PANoptosome, shares key characteristics of apoptosis, pyroptosis, and necroptosis but cannot be accounted for by any of them alone^6^. Further, crosstalk among the three PCD types facilitates the conversion from one mode to another, under certain conditions^7–9^. Therefore, therapeutic strategies trigging PANoptosis may provide an alternative way against cancer, especially when apoptosis is inhibited.

As an inflammatory PCD, PANoptosis is generally associated with a cytokine storm and plays negative roles in inflammatory or infectious diseases^10^. In the context of cancer, it is believed that PANopotosis could be beneficial. For example, the combination of TNF-α and IFN-γ can induce inflammatory cell death, i.e., PANoptosis, in different human cancer cell lines and suppress the growth of tumors in vivo^11^, suggesting that PANoptosis might be an attractive therapeutic target. Evidence indicated that pyroptosis, necroptosis, and ferroptosis are tightly associated with antitumor immunity^12^. However, the association between PANoptosis as a whole and anticancer immunity remains unclear. In addition, the function and regulation pattern of PANoptosis components have not been comprehensively elucidated so far.

In this study, we performed a systematic bioinformatics analysis of the PANoptosome complex to investigate its roles and underlying mechanisms in pan-cancer. We evaluated the expression, genomics, epigenomics, and the prognostic value of PANoptosome across 33 cancer types. Moreover, we calculated the PANoptosis score and further assessed its association with the tumor immune landscape, including the tumor microenvironment (TME), immune cell infiltration, and immunotherapy responses. Our results reveal the potential role of the PANoptosome in various cancers, which may facilitate the discovery of novel prognostic and immunotherapeutic biomarkers.

## Materials and methods

### Data collection and processing

RNA sequencing data and clinical information on 33 tumors and normal tissues (Supplementary Table S1) were retrieved from The Cancer Genome Atlas (TCGA) and the Genotype-Tissue Expression (GTEx) using USCS Xena (https://xenabrowser.net). The "RMA" package^13^ was used to remove not available and duplicates in the expression data; the data were then log_2_ (transcripts per kilobase million (TPM) + 1) converted for further analysis. Ten genes including NLRP3, CASP1, CASP8, TNFAIP3, RIPK1, RIPK3, NR2C2, RBCK1, PSTPIP2, and PYCARD (Supplementary Table S2) were identified as components of the PANoptosome, according to a previous study^10^.

The data of immune cell infiltration were downloaded from the Tumor Immune Estimation Resource **(**TIMER**)** (http://timer.cistrome.org) and Immune Cell Abundance Identifier (ImmuCellAI) databases (http://bioinfo.life.hust.edu.cn/ImmuCellAI).

The dataset GSE35640 (65 metastatic melanoma patients treated with MAGE-A3 immunotherapy)^14^, GSE 91061 (65 melanoma patients treated with Anti-CTLA4 and ant-PD1)^15^, and GSE135222 (27 advanced non-small cell lung carcinoma patients treated with anti-PD-1/PD-L1)^16^ were downloaded from the Gene Expression Omnibus database (https://www.ncbi.nlm.nih.gov) to explore the prediction ability of immunotherapy response based on PANoptosis scores.

### Gene set cancer analysis using GSCALite

The Gene Set Cancer Analysis (GSCALite) server offers an integrated database for gene set analysis, including genomic variation, pathway, and drug sensitivity analysis (http://bioinfo.life.hust.edu.cn/web/GSCALite/)^17^. In this study, the genomic alterations (mutation, copy number variation (CNV), and methylation) associated with the PANoptosome were analyzed using GSCALite.

### PANoptosis score analysis

The single sample gene set enrichment analysis (ssGSEA) algorithm of the R package ‘GSVA’^18^ was used to compute the PANoptosis scores of each sample in each cancer type. The score reflects the overall expression level of the PANoptosome.

### Survival analysis

Survival information associated with the tumor samples was retrieved from TCGA. Univariate Cox regression (uniCox) analysis was conducted to test the association of PANoptosome expression with survival using the R packages ‘Survival’ and ‘Survminer’^19^. We further explored the predictive value of PANoptosis scores for survival, including overall survival (OS), disease-specific survival (DSS), disease-free interval (DFI), and progression-free interval (PFI).

### Gene enrichment analysis

Gene set variation analysis (GSVA) was performed to identify biological pathways associated with the PANoptosis score. We used the R package ‘GSVA’^18^ to compute pathway scores of HALLMARK term downloading from the MSigDB database (http://software.broadinstitute.org/gsea/msigdb/index.jsp). Correlations between PANoptosis scores and these pathways were tested.

### TME and immune cell infiltration analyses

The ESTIMATE algorithm was used to calculate the immune score, stromal score, and tumor purity of each sample across different cancer types using the R package ‘estimate’^20^. The correlation between the PANoptosis score and these scores was analyzed using Spearman’s correlation test. In addition, we evaluated the correlation between PANoptosis score and TME-related pathways according to a previous study^21^. The associations of PANoptosis score with the abundance of immune cells as well as immune-related genes were further analyzed through Spearman’s correlation tests.

### Human Protein Atlas

Human Protein Atlas (HPA) (https://www.proteinatlas.org), is a website containing the immunohistochemistry-based expression of tumor tissues and normal tissues^22^. In the present study, we compared the protein level of PYCARD in human normal and three tumor tissues including kidney renal clear cell carcinoma (KIRC), glioblastoma (GBM), and pancreatic adenocarcinoma (PAAD) by downloading immunohistochemical images from the HPA.

### Cell lines and cell culture

Human normal renal tubular epithelial cells (HK-2), human GBM cell lines (U87 MG, T98G), human pancreatic duct epithelial cell line (hTERT-HPNE), and human PAAD cell line (PANC-1) were cultured in Dulbecco's modified Eagle's medium (Gibco, United States) containing 10% Fetal Bovine Serum (FBS, Gibco, United States) and 1% penicillin/streptomycin. Human KIRC cell lines (786-O, CaKi-1), human normal astrocytes (HA1800), and human PAAD cell line (ASPC-1) were cultured in RPMI-1640 medium (Gibco, United States) containing 10% and 1% penicillin/streptomycin. Cells were cultured in incubator containing 5% CO_2_ at 37 °C.

### Quantitative Real-time PCR analysis

After all cell lines being passaged for three generations, total RNA was isolated from cells using Trizol reagent (Invitrogen Carlsbad, CA, USA). The total RNA (1ug) was reversed transcribed using a PrimeScript RT Master Mix (TaKaRa Biotechnology, Otsu, Japan). Quantitative Real-time PCR was conducted using SYBR Premix EsTaq (Mei5 Biotechnology, Beijing, China) and run in real-time PCR detection system (Applied Biosystems, Foster City, CA, USA). The primer sequences were as follows: PYCARD (forward:AGCTCACCGCTAACGTGCTGC,reverse:GCTTGGCTGCCGACTGAGGAG),GAPDH(forward:GGAAGCTTGTCATCAATGGAAATC,reverse:TGATGACCCTTTTGGCTCCC).

### Statistical analysis

Differences between groups were estimated using Student’s *t*-test. Spearman’s test was adopted to analyze the correlation between two variables. All the statistical analyses were performed using R software (https://www.r-project.org/, version 4.0.3). Statistical significance is reported at *P* < 0.05.

## Results

### Expression and prognostic value of the PANoptosome

**Figure 1:**
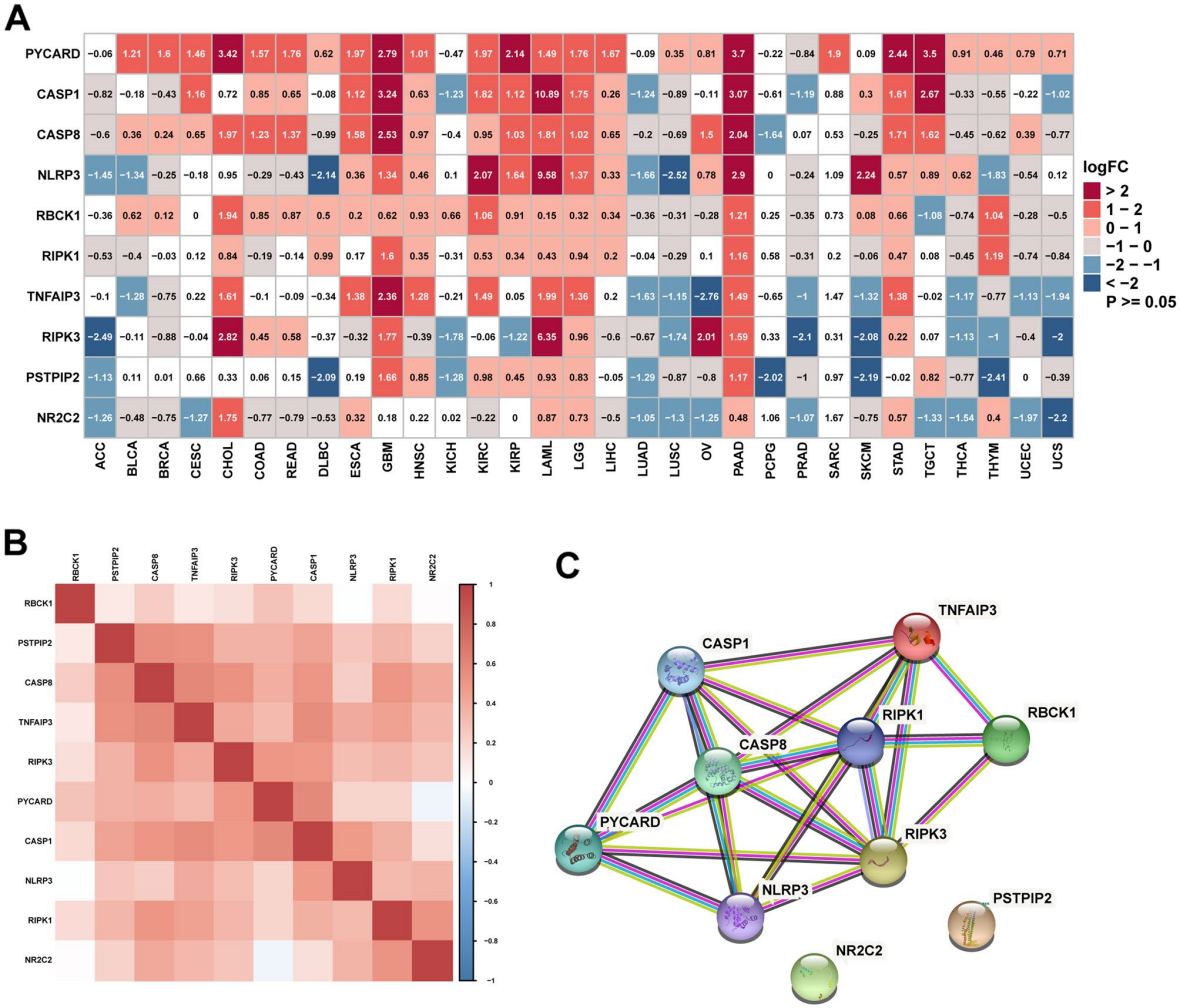
The expression of PANoptosome components. (**A**) The differential expression of PANoptosome components between tumor and normal tissues in pan-cancer based on TCGA and GTEx databases. Red represents high expression, and blue represents low expression in tumor. *P *value and logFC are presented. (**B**) The correlation analysis of PANoptosome members in TCGA database. Darker shades mean a stronger correlation. (**C**) Protein–protein interaction (PPI) network of PANoptosome.

First, we analyzed the differential expression of PANoptosome components between tumor and normal samples by combing the data from the TCGA and GTEx databases in pan-cancer. Heterogeneous expression patterns of PANoptosis genes were observed across different cancer types (Fig. 1A). Among these genes, PYCARD was upregulated in most cancers. In addition, PANoptosis genes were significantly upregulated in cholangiocarcinoma (CHOL), GBM, acute myeloid leukemia (LAML), lower grade glioma (LGG), PAAD, and stomach adenocarcinoma (STAD), while their levels were downregulated in adrenocortical carcinoma (ACC), lung adenocarcinoma (LUAD), lung squamous cell carcinoma (LUSC), prostate adenocarcinoma (PRAD), and uterine carcinosarcoma (UCS). However, most genes did not present a significant difference in pheochromocytoma, paraganglioma (PCPG), and sarcoma (SARC). The correlation among PANoptosis genes was analyzed based on pan-cancer samples, and positive correlations were observed between these genes (Fig. 1B). Figure 1C shows the interaction network of these regulators as visualized using the STRING online website (https://string-db.org/).

**Figure 2:**
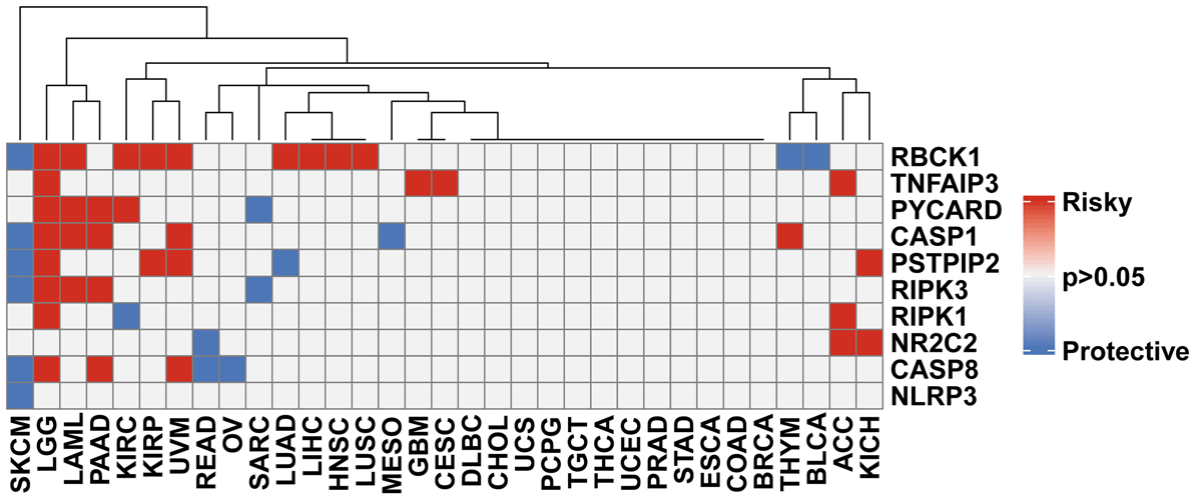
The prognostic significance of PANoptosome components.

To determine the correlation of PANoptosis genes with survival, we performed uniCox regression analysis across 33 cancer types (Fig. 2). The results indicated that most PANoptosis genes were tumor risk factors. Among them, RBCK1 was associated with the highest number of cancer types. In addition, for skin cutaneous melanoma (SKCM), six of ten PANoptosis components predicted better survival, whereas eight of ten genes were predictive of poor prognosis in LGG.

### Experiments to validate the expression of PYCARD

**Figure 3:**
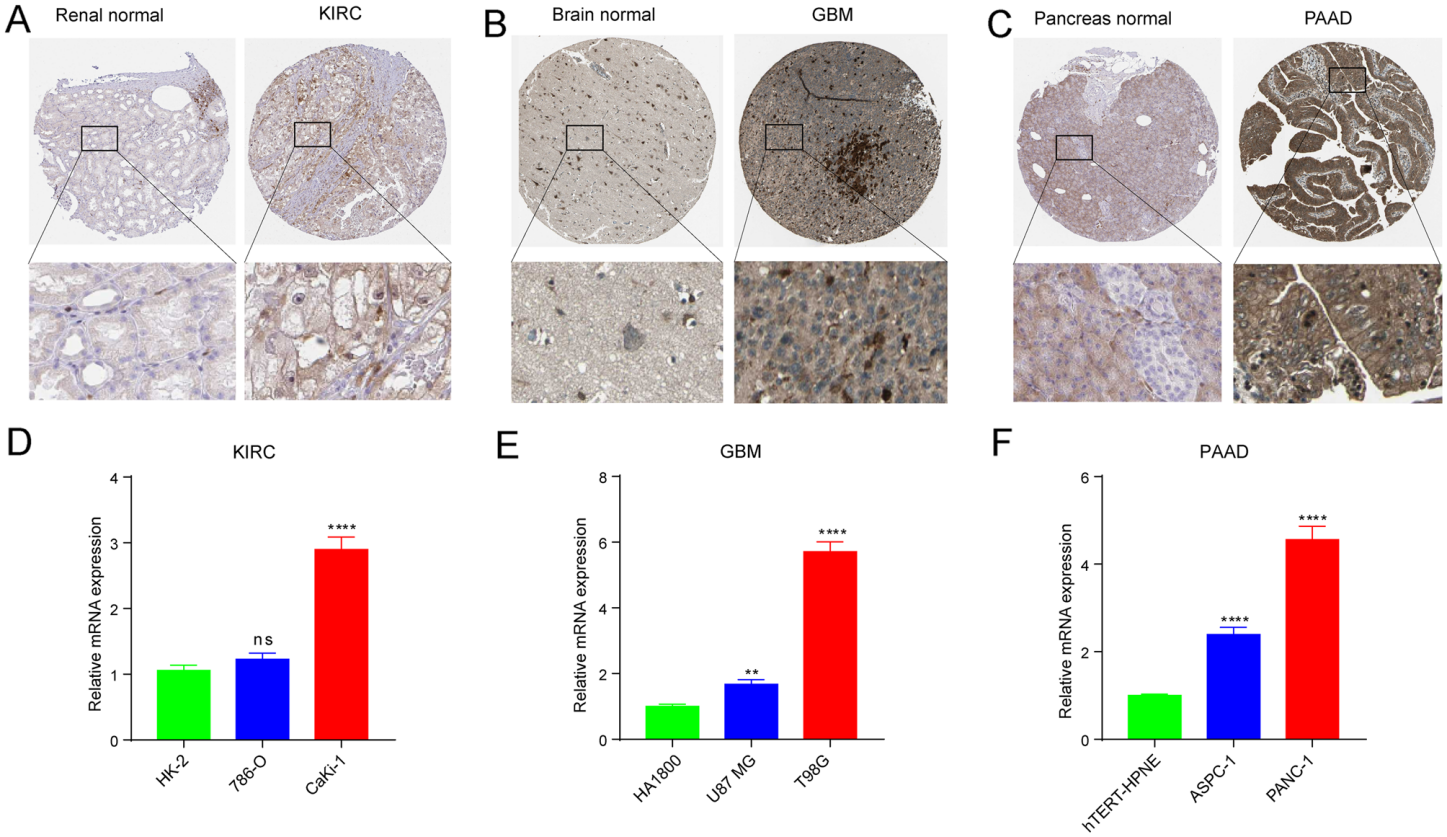
Protein and mRNA expression levels of PYCARD. (**A**–**C**) The protein expression of PYCARD in (**A**) kidney renal clear cell carcinoma (KIRC); (**B**) glioblastoma (GBM); (**C**) pancreatic adenocarcinoma (PAAD). (**D**–**F**) The mRNA expression of PYCARD in (**D**) KIRC cells; (**E**) GBM cells; (**F**) PAAD cells.

Based on the expression and survival analysis of PANoptosis genes in pan-cancer, we further validated the expression level of PYCARD in KIRC, GBM, and PAAD. For protein level from HPA database, we observed a much stronger staining of PYCARD in three tumor tissues compared to corresponding normal tissues (Fig. 3A–C). Consistent with the result of immunohistochemistry, PYCARD was significantly up-regulated in human KIRC cells (CaKi-1), GBM cells (U87 MG and T98G), and PAAD cells (ASPC-1 and PANC-1) (Fig. 3D–F).

### Analysis of genetic alteration

**Figure 4:**
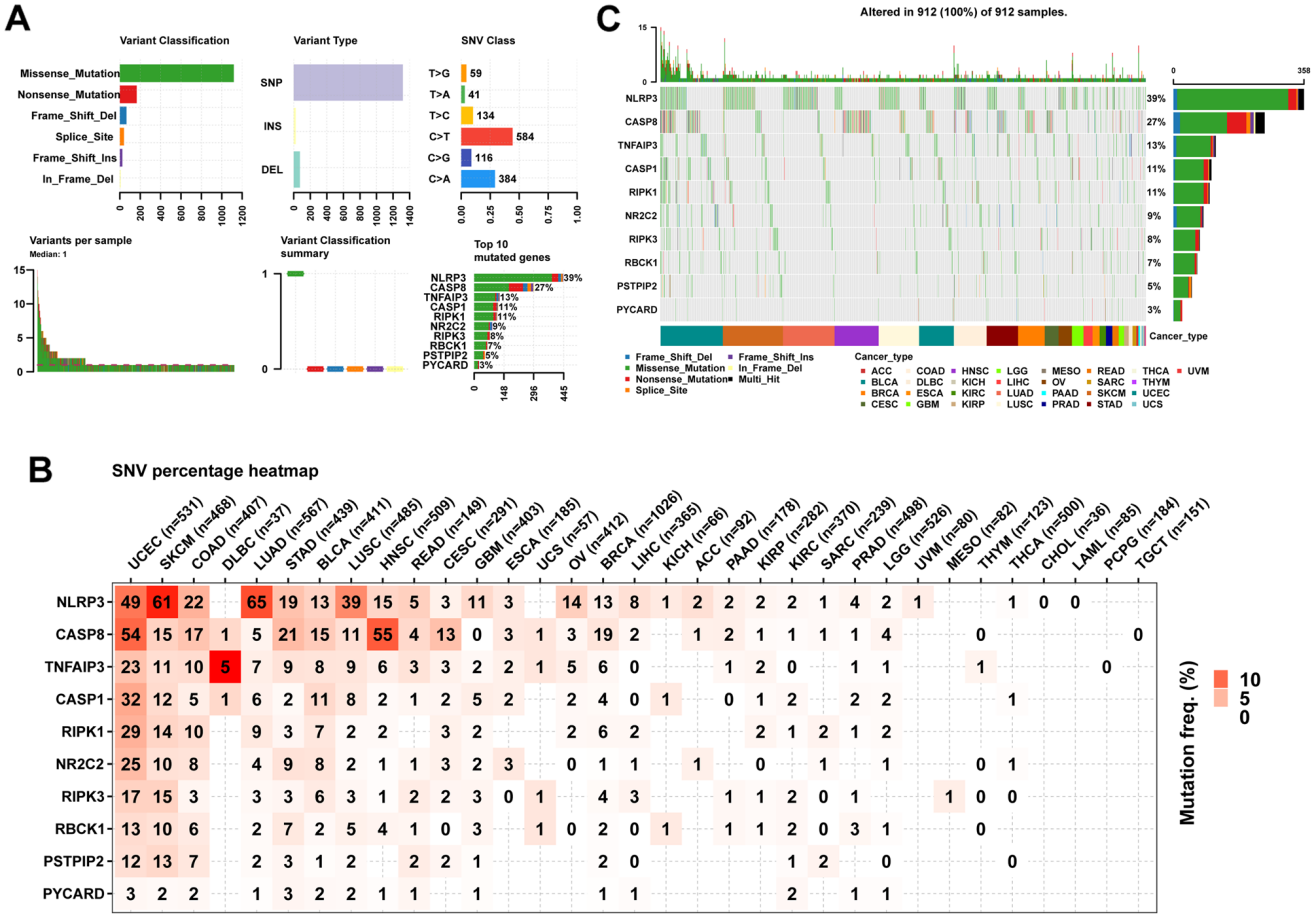
The SNV alteration of PANoptosis genes in pan-cancer. (**A**) The summary of SNV alteration. (**B**) SNV frequency of genes in indicated cancer types. (**C**) The waterfall plot shows the mutation distribution of 10 mutated genes across different cancers.

Tumorigenesis and progression are strongly associated with genomic changes. We assessed single nucleotide variants (SNV), CNV, and methylation alterations of 10 PANoptosis genes in cancer patients using the GSCALite database. Figure 4A shows the variant classification, variant type, SNV class, variants per sample, variant classification summary, and the frequency of 10 mutated genes in pan-cancer samples. SNV frequency analysis indicated that NLRP3 had the highest SNV percentage (65%) in LUAD (Fig. 4B), and the SNV classification of missense mutation was the predominant type in pan-cancer. The top three mutated genes were NLRP3 (39%), CASP8 (27%), and TNFAIP3 (13%), whereas PYCARD exhibited the lowest mutation frequency (3%) (Fig. 4C).

**Figure 5:**
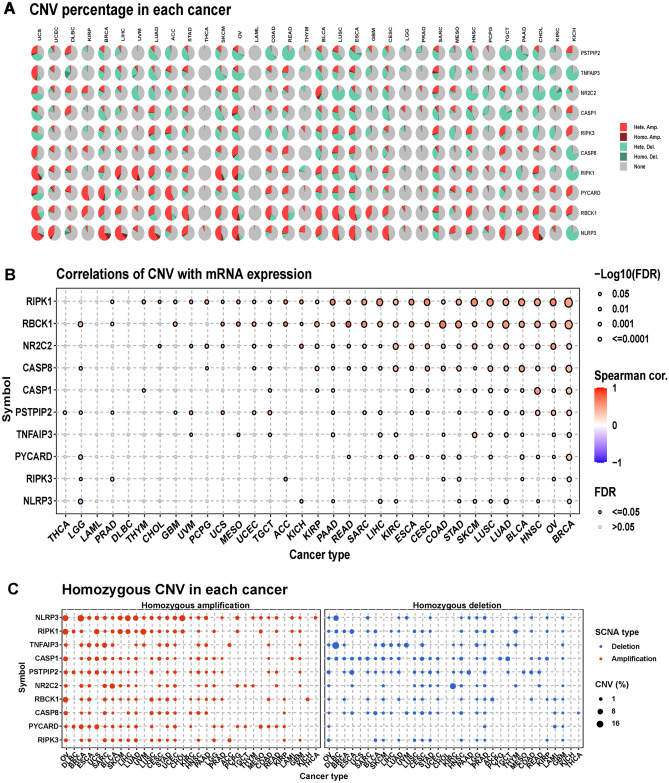

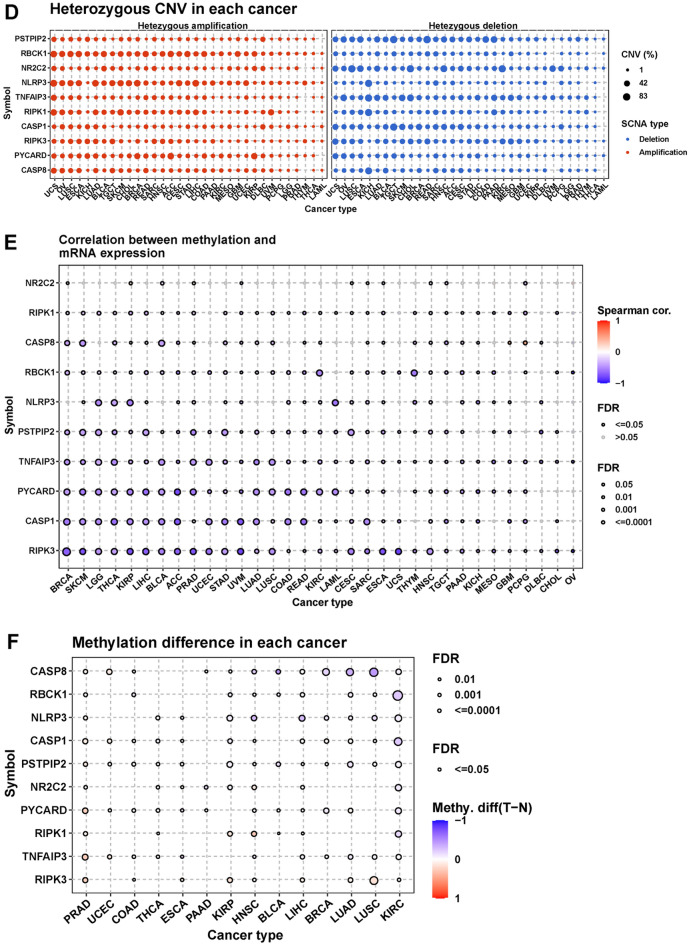
The CNV and methylation levels of PANoptosis genes in pan-cancer. (**A**) Pie plot displays the global CNV profile of PANoptosis genes in 33 cancer types. (**B**) The correlation between CNV and genes mRNA expression in TCGA pan-cancer samples. (**C**) The profile of homozygous CNV shows the percentage of amplifications and deletions of homozygous CNV for genes in each cancer. (**D**) The profile of heterozygous CNV shows the percentage of amplifications and deletions of heterozygous CNV for genes in each cancer. (**E**) Methylation difference between tumor and normal samples in indicated tumor types. (**F**) The correlation between methylation and genes mRNA expression in TCGA pan-cancer samples.

CNV types and the proportion of each CNV type in each cancer type are shown in Fig. 5A. Heterozygous amplification and deletion were the primary CNV types. Correlation analysis indicated that the CNVs of PANoptosis genes were mainly positively associated with their mRNA expression, especially in PIPK1 and RBCK1 (Fig. 5B). PANoptosis genes had different levels of CNV alterations in each cancer type, particularly regarding heterozygous CNV changes (Fig. 5C,D). We further conducted methylation analysis, indicating that the methylation levels of 10 components were inversely correlated with mRNA expression in general (Fig. 5E). Figure 5F shows the difference in methylation levels in PANoptosis members between normal and tumor tissues, and the differential results varied markedly among cancers.

### Differential comparison of PANoptosis scores

**Figure 6:**
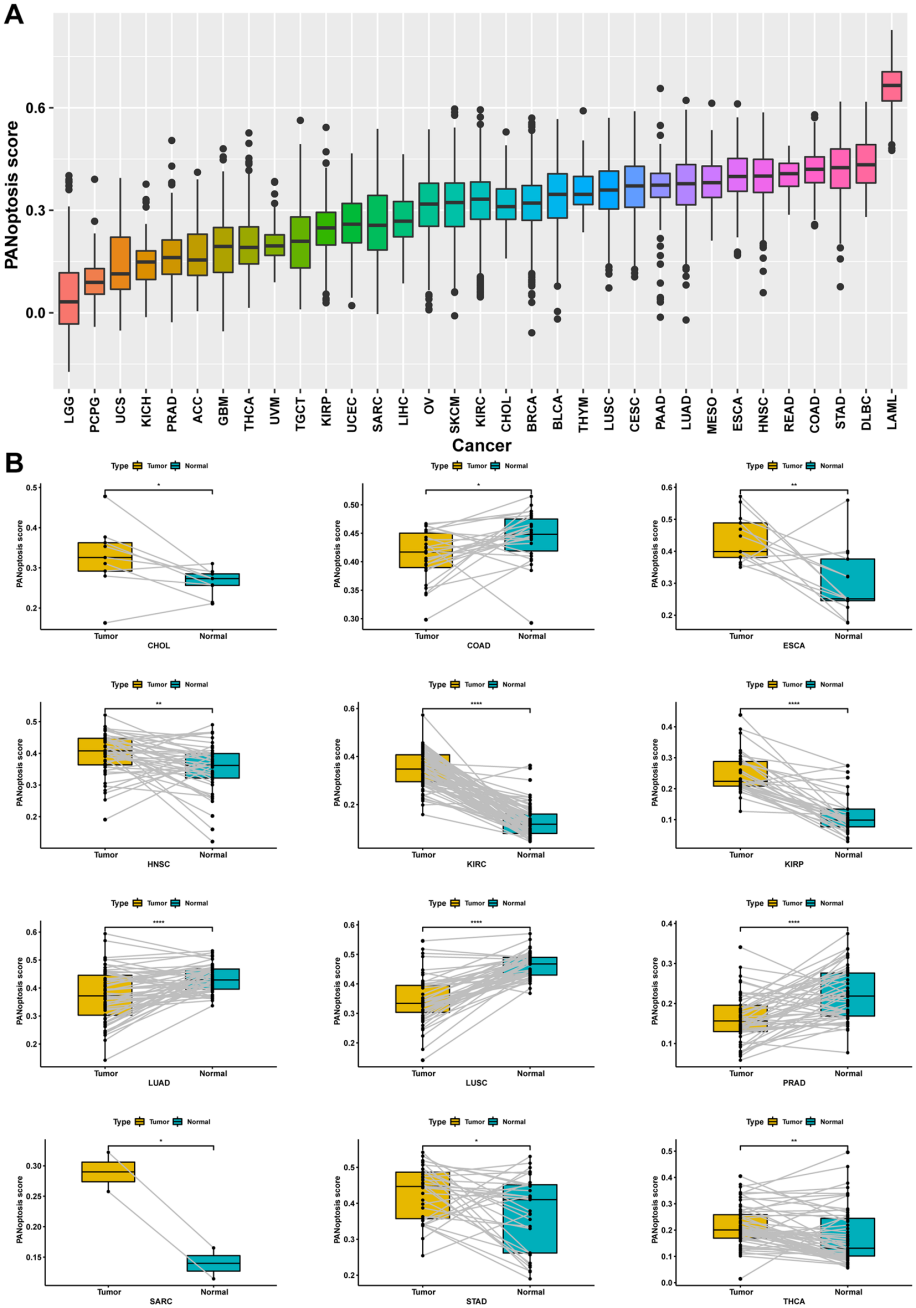
The distribution of PANoptosis score. (**A**) The PANoptosis score in each cancer type. (**B**) Comparison of the PANoptosis scores in tumor and corresponding normal tissues based on TCGA cohort.

The PANoptosis score in pan-cancer was calculated using the ssGSEA approach based on the TCGA database. The highest PANoptosis score was found in LAML, while the lowest was observed in LGG (Fig. 6A). For tumor and adjacent normal tissues, the PANoptosis score was higher in CHOL, esophageal carcinoma (ESCA), head and neck squamous cell carcinoma (HNSC), KIRC, kidney renal papillary cell carcinoma (KIRP), SARC, STAD, and thyroid carcinoma (THCA), and it was lower in colon adenocarcinoma (COAD), LUAD, LUSC, and PRAD (Fig. 6B).

### Survival analysis of PANoptosis scores

**Figure 7:**
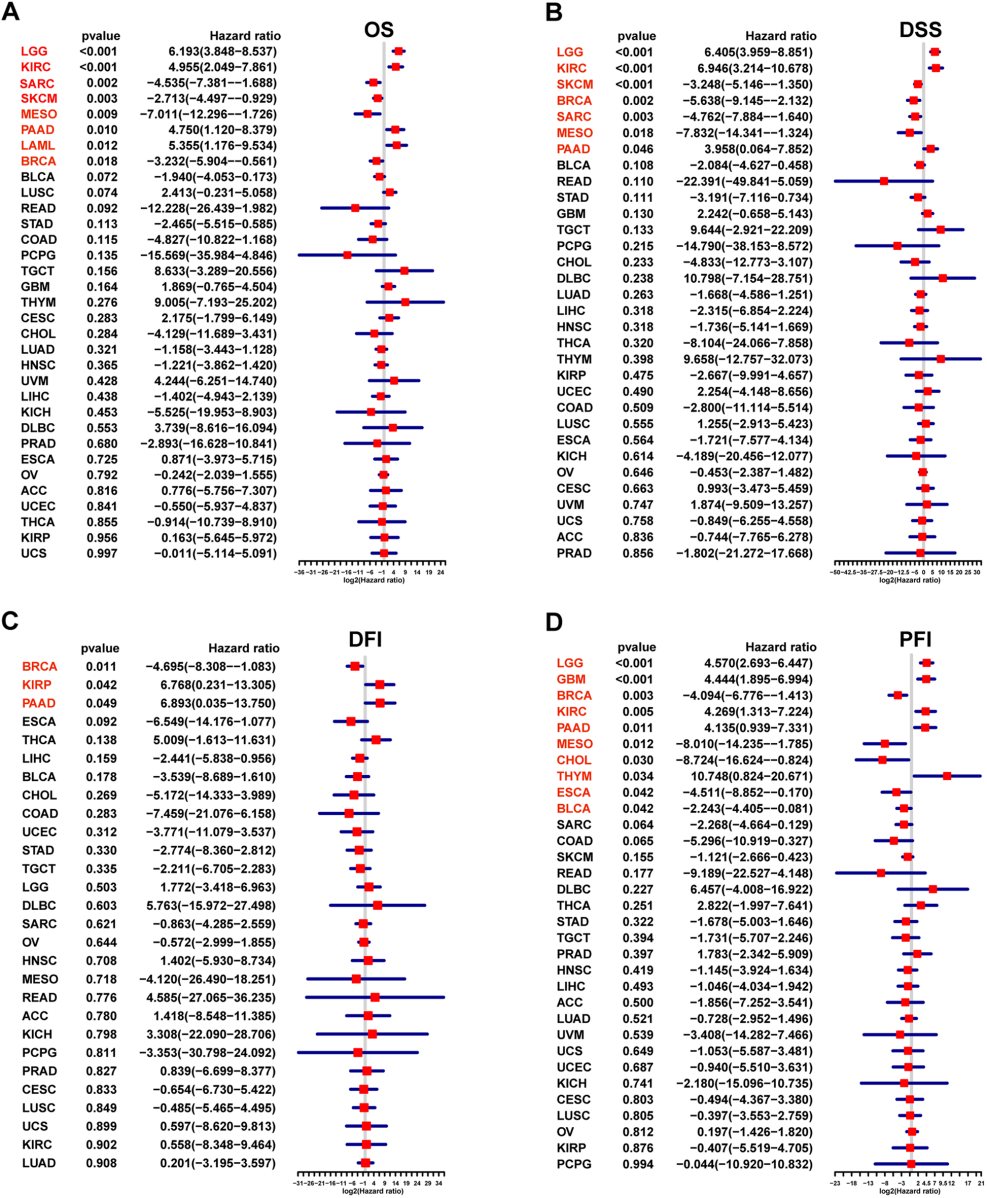
The association between PANoptosis score and survival prognosis in pan-cancer. (**A**) Overall survival. (**B**) Disease-specific survival. (**C**) Disease-free interval. (**D**) Progression-free interval.

The association between PANoptosis scores and cancer survival outcomes was further investigated with regard to OS, DSS, DFI, and PFI. uniCox analysis indicated that the PANoptosis score was a risk factor for OS in LGG, KIRC, PAAD, and LAML, and it was a protective factor in SARC, SKCM, mesothelioma (MESO), and breast invasive carcinoma (BRCA) (Fig. 7A). DSS analysis suggested that PANoptosis score was a risk factor for patients with LGG, KIRC, and PAAD, and it was a protective factor in SKCM, BRCA, SARC, and MESO (Fig. 7B). DFI analysis showed that the PANoptosis score was as a risk factor in KIRP and PAAD, while as a protective factor only in BRCA patients (Fig. 7C). Finally, the PFI analysis indicated that PANoptosis score was a risk factor in LGG, GBM, KIRC, PAAD, and thymoma (THYM). And it was a protective factor for patients with BRCA, MESO, CHOL, ESCA, and bladder urothelial carcinoma (BLCA) (Fig. 7D).

### Functional enrichment analysis of PANoptosis scores

**Figure 8:**
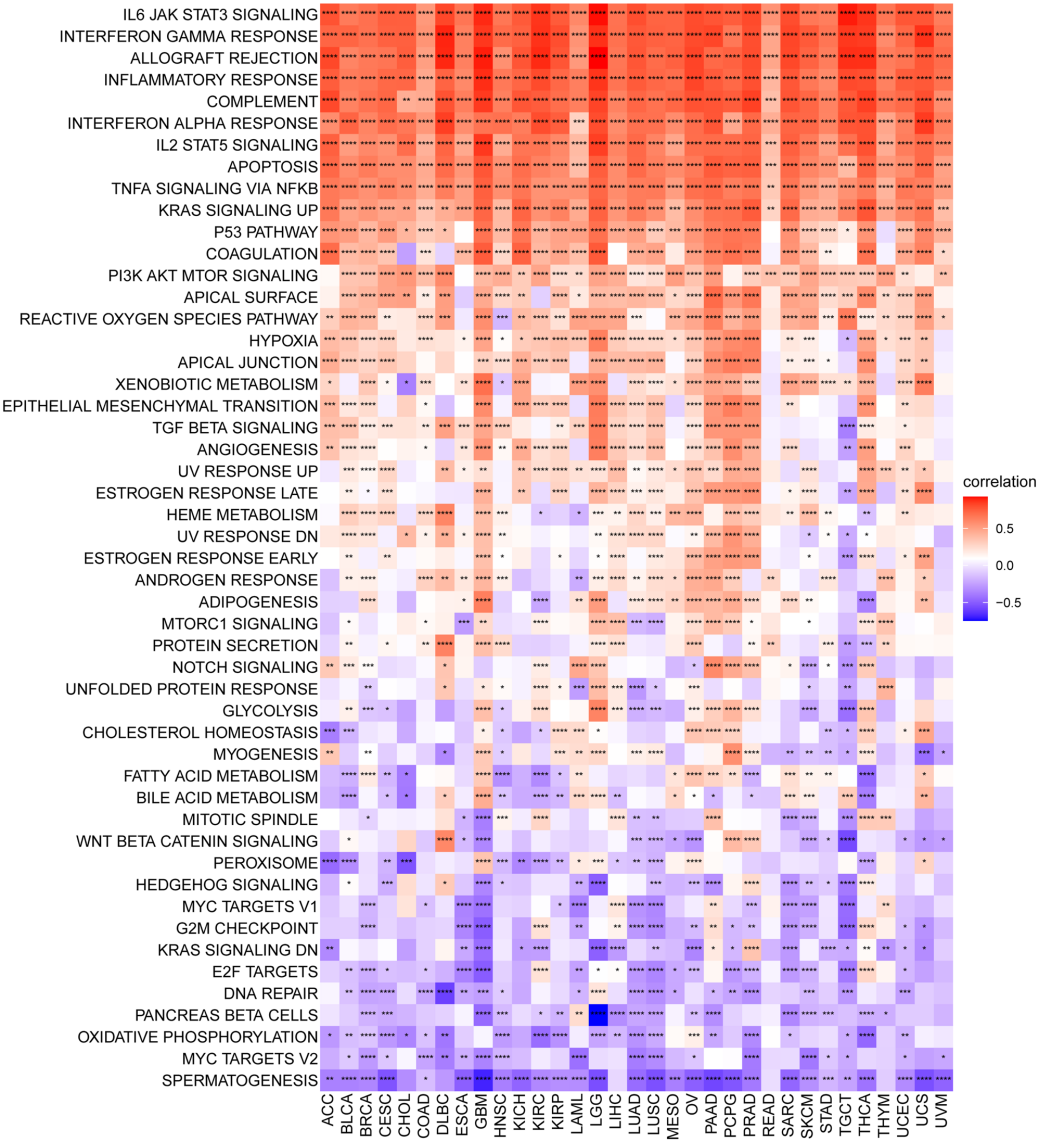
The correlation of PANoptosis score with HALLMARK pathways in pan-cancer.

To explore the function of PANoptosis scores in different cancers, we identified the potential pathways associated with PANoptosis scores through GSVA based on the HALLMARK terms. The PANoptosis score was positively correlated with many malignant pathways in pan-cancer, especially IL6-JAK-STAT3 signaling, interferon-gamma response, inflammatory response, IL2-STAT5 signaling, and TNF-a signaling via NF-kB signaling (Fig. 8). These results suggest that the PANoptosis score markedly affects the inflammatory response and the tumor immune microenvironment.

### PANoptosis scores are associated with TME and immune cell infiltration

**Figure 9:**
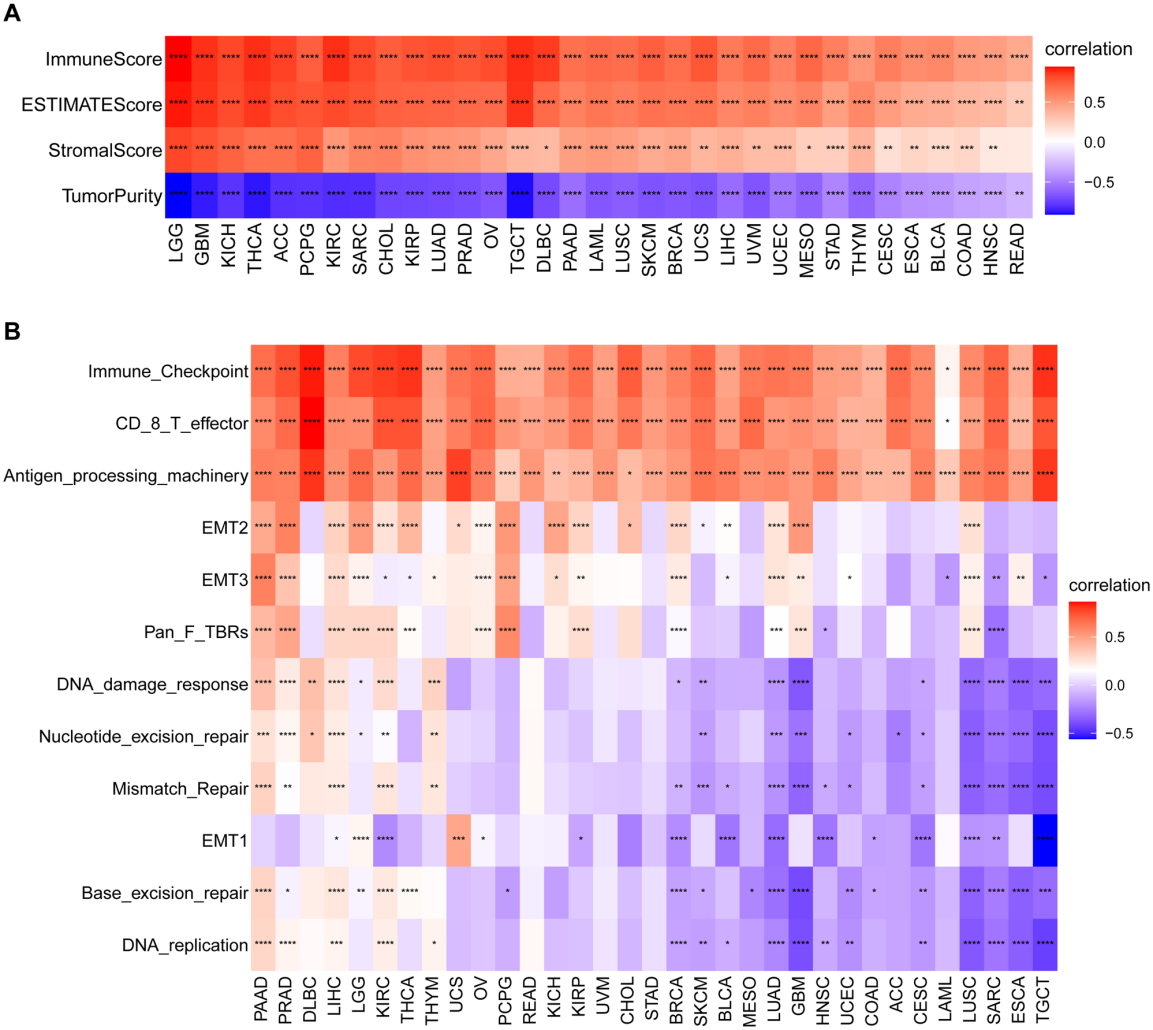
TME analysis of PANoptosis score in pan-cancer. (**A**) Heatmap shows the correlation between PANoptosis score and ImmuneScore, ESTIMATEScore, StromalScore, and TumorPurity. (**B**) Heatmap shows the correlation between PANoptosis score and TME-related pathways. *P < 0.05, **P < 0.01, ***P < 0.001, ****P < 0.0001.

Increasing evidence suggested that the TME plays a critical role in multidrug resistance, tumor progression, and metastasis^23^. To explore the connection between PANoptosis and the TME, we applied the ESTIMATE algorithm to evaluate the correlation of PANoptosis scores with the composition of TME. The results indicated that the PANoptosis score was positively correlated with the immune, stromal, and ESTIMATE scores in pan-cancer (Fig. 9A). Furthermore, we investigated 12 TME-related pathways based on published data, including immune, stromal, and DNA repair-related pathways. The PANoptosis score was strongly correlated with immune-related pathways, such as immune checkpoint, CD8 T effector, and antigen processing machinery pathways (Fig. 9B).

**Figure 10:**
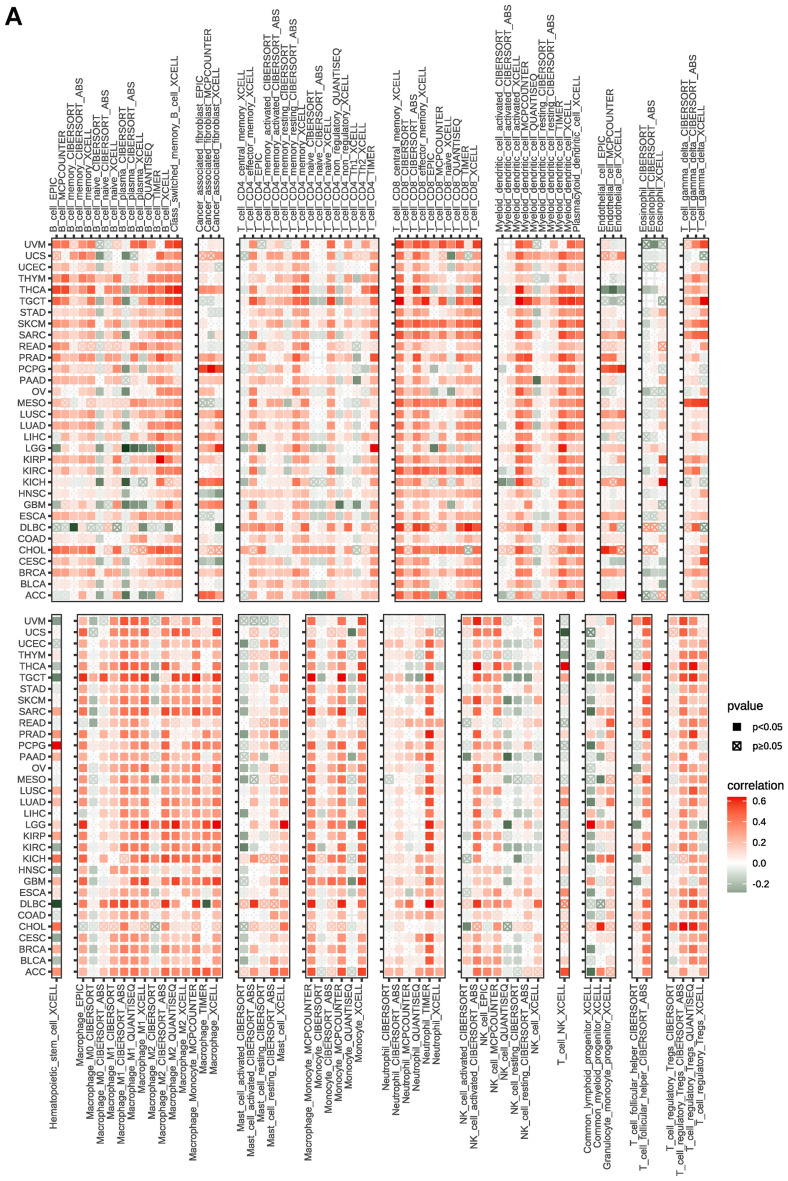

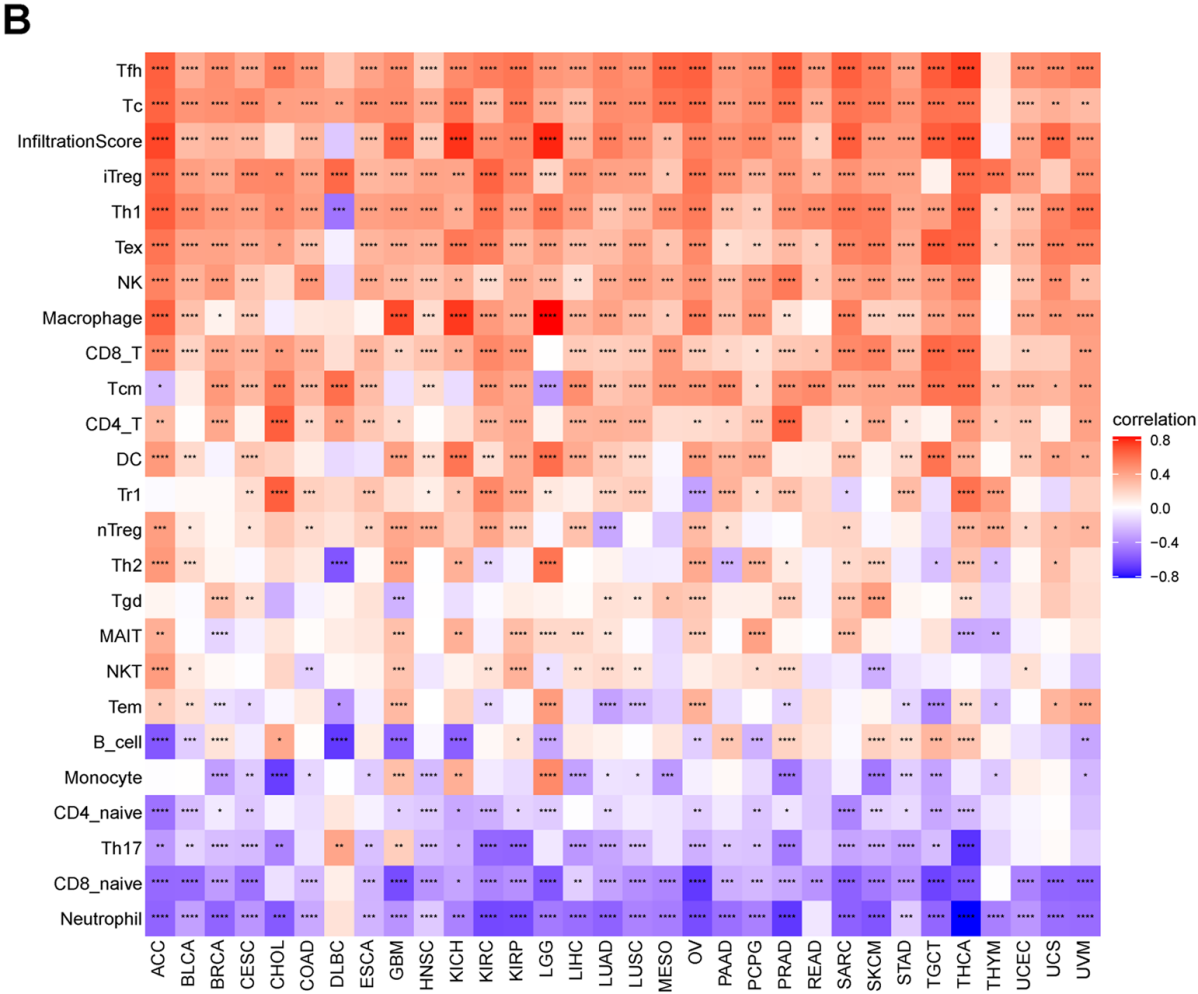
Relationship of PANoptosis score with immune cell infiltration analysis. (**A**) The analysis of immune cell infiltration based on TIMER2 database. (**B**) The analysis of immune cell infiltration based on ImmuCellAI database.

To clarify the relationship between PANoptosis scores and immune cell infiltration in the TME, we conducted correlation analysis in pan-cancer. The results based on the TIMER database indicated that the PANoptosis score was strongly correlated with most immune cells in pan-cancer (Fig. 10A). Consistent with the above results, the same trend was observed from the data of the ImmuCellAI database. It showed that the PANoptosis score was positively correlated with the infiltration levels of NK cells, CD8+ T cells, CD4+ T, DC cells, and macrophages, and it was negatively correlated with the levels of neutrophil cells, naive CD4+ T cells, and naïve CD8+ T cells (Fig. 10B).

**Figure 11:**
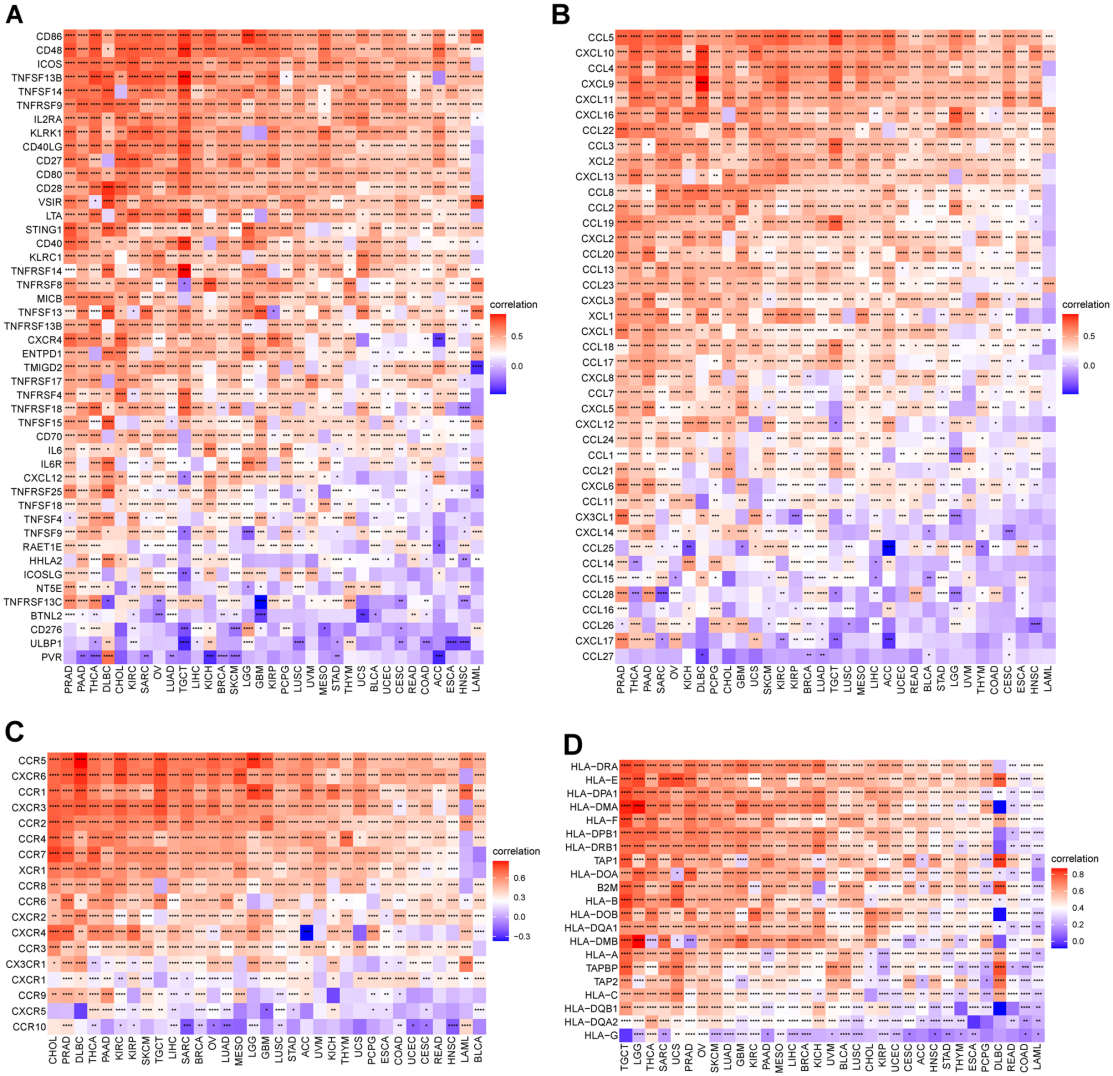
Correlation between PANoptosis score and immune associated genes. (**A**) immune activated genes. (**B**) Chemokines. (**C**) Chemokine receptors. (**D**) MHC genes in pan-cancer. *P < 0.05, **P < 0.01, ***P < 0.001, ****P < 0.0001.

In addition, we investigated the correlation of PANoptosis scores and immune-related genes, including immune-activation genes, chemokines, chemokine receptors, and MHC genes. Most immunomodulators exhibited a strong positive correlation with PANoptosis scores in pan-cancer (Fig. 11).

### PANoptosis scores are associated with immunotherapy responses

**Figure 12:**
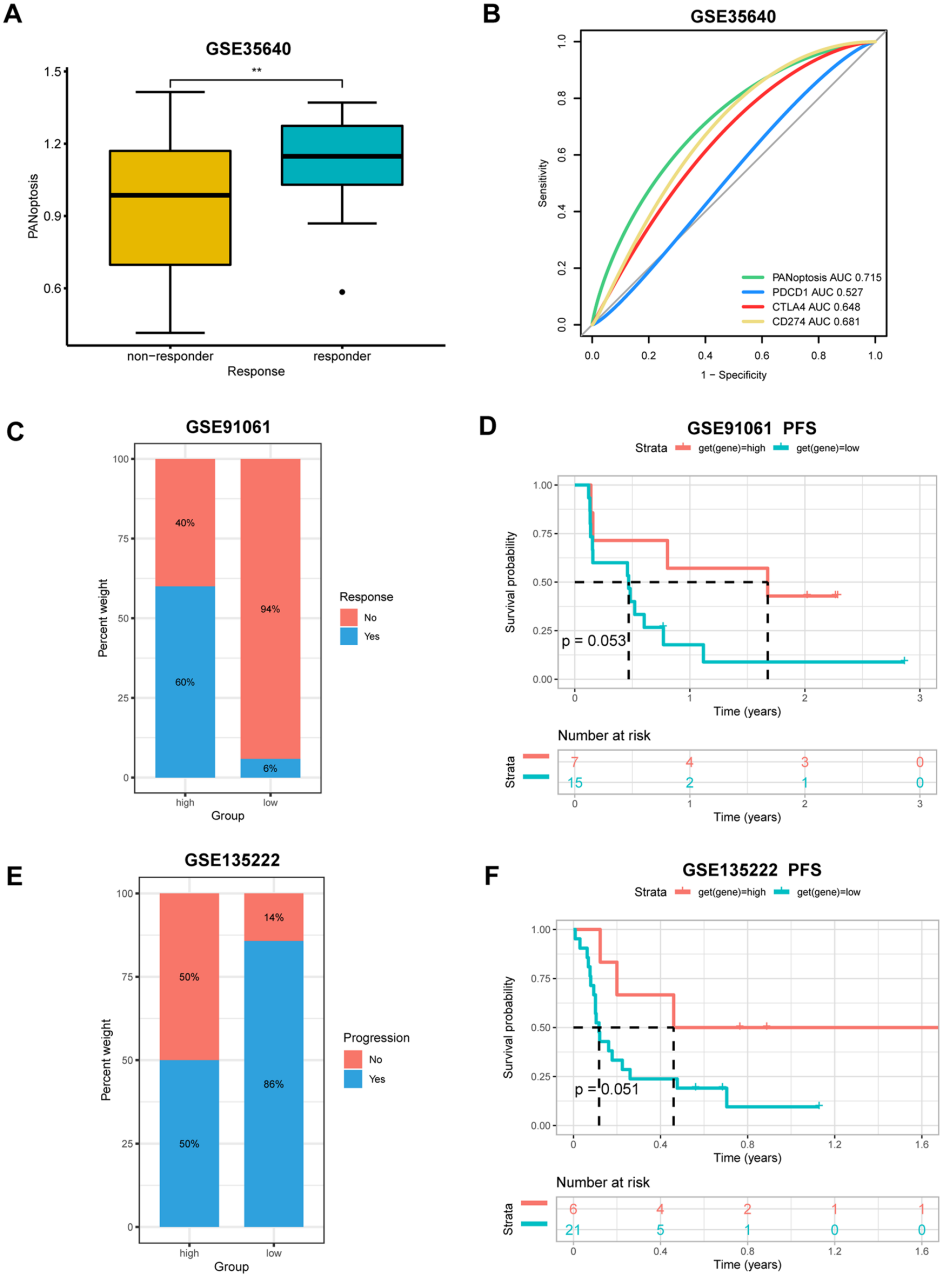
The correlation between PANoptosis score and immunotherapy response. (**A**) The difference of PANoptosis score between immunotherapy responder and non-responder in GSE35640 cohort. (**B**) ROC curve analysis for evaluating the predictive performance on immunotherapy response. (**C**,**E**) The percentage of responsive and progressive patients in high- and low- PANoptosis score groups in (**C**) GSE91061; (**E**) GSE13522 cohorts. (**D**,**F**) The Kaplan–Meier PFS analysis of PANoptosis score in (**C**) GSE91061 and (**E**) GSE13522 cohorts. PFS, progression-free survival. **P < 0.01.

Based on the established link between PANoptosis and immunity, we speculated that patients with high PANoptosis scores should be sensitive to immunotherapy. To confirm this, we retrieved information on immunotherapy responses and computed PANoptosis scores of patients in three cohort. We discovered that patients in the immunotherapy responder group had significantly higher PANoptosis scores than the non-responder group (Fig. 12A). Furthermore, we assessed the area under the curve (AUC) for predicting the immunotherapy responses with respect to PANoptosis scores, PD-1, PD-L1, and CTLA-4 levels. The receiver operating characteristics curve showed that the AUC of PANoptosis score (0.715) was larger than that of three immune checkpoints, which indicated that PANoptosis scores had a higher capacity to predict immunotherapy responses (Fig. 12B). The results of other two datasets suggested that there was higher percentage of responsive patients in high PANoptosis scores group (Fig. 12C,E). Meanwhile, patients with high PANoptosis scores showed longer PFS, even though they were not statistically significant (*p* > 0.05) (Fig. 12D,F).

## Discussion

PANoptosis, as a newly discovered type of PCD, has the potential to overcome apoptosis resistance and prime the immune system by inducing inflammatory cell death in tumors^24,25^. Given its critical role in cancer, a better understanding of the PANoptotic master regulators is necessary for developing PANoptosis-targeted approaches. Using previously published data, we provided a comprehensive and systematic description of PANoptosis genes across 33 various cancer types. Our results revealed the potential mechanisms of PANoptosis genes and the associated pathways of common PANoptosome in cancers, thus elucidating the overall regulation of the PANoptosome complex in pan-cancer.

In the present study, we first evaluated the expression, prognostic value and genomic landscape of PANoptosis components. Our results indicated that PANoptosis genes were aberrantly expressed and associated with patient survival in a cancer-type-dependent manner. Specifically, most PANoptosis genes served as tumor risk factors in pan-cancer. Through genetic analyses, we observed that gene mutation was remarkable in NLRP3, CASP8, and TNFAIP3. It has been reported that cells with CASP8 mutation are resistant to extrinsic apoptosis^26^, and mutation in CASP8 prevents cytolytic T cells from killing tumor cells by FasL-Fas interactions, representing a mechanism of immune evasion^27^. Regarding CNV analysis, we found that PANoptosis genes, especially NLRP3, RBCK1, PSTPIP2, and TNFAIP3, may promote tumor progression via CNV alteration. For example, RBCK1 was highly upregulated in HNSC and LUSC and was associated with poor survival in these two cancer types. In addition, many reports have explored the oncogenic role of RBCK1 in different cancers^28–30^. The NLRP3 inflammasome is a key component of the innate immune system and consists of NLRP3, PYCARD/ASC, and pro-CASP1^31^. NLRP3 activation in tumor-associated macrophages can regulate their polarization, which increases lung metastasis of PDAC^32^. Inhibition of NLRP3 inflammasome decreases cell invasiveness in HNSCC^33^. These results suggested that PANoptosis genes play a crucial role in tumor progression.

To further infer the activity of PANoptosome, we calculated the PANoptosis score for each patient in the pan-cancer dataset. We found that the PANoptosis score was higher in CHOL, ESCA, HNSC, KIRC, KIRP, SARC, STAD, and THCA, and it was lower in COAD, LUAD, LUSC, and PRAD, compared to adjacent normal tissues. The result of uniCox analysis showed that PANoptosis score was a risk factor in LGG, KIRC, PAAD, LAML, GBM, and THYM, and it acted as a protective factor in SARC, SKCM, MESD, BRCA, MESO, CHOL, ESCA, and BLCA, which was consistent with previous results (Fig. 2). In the pathway analysis, we found that PANoptosis score was positively correlated with IL6-JAK-STAT3 signaling, interferon-gamma response, inflammatory response, IL2-STAT5 signaling, and TNF-a signaling via the NF-kB signaling pathway in 33 diverse cancer types. These results indicated that PANoptome was a key regulator of immune and inflammatory responses.

Increasing evidence has demonstrated that immune status in tumors is strongly influenced by the composition and infiltrating levels of cells in the corresponding environment^34,35^. Therefore, we examined the correlation of PANoptosis score with TME as well as the infiltration level of immune cells. As a result, a positive correlation of PANoptosis score with the immune, stromal, and ESTIMATE scores was observed in pan-cancer. Moreover, we found that the PANoptosis score was closely correlated with most immune cells in pan-cancer. To further explore the role of PANoptosis genes in cancer immunity, we analyzed the correlation of PANoptosis score with a series of immunomodulators which are important for immunotherapy^36^. The result showed that most immune regulators had a positive association with the PANoptosis score in pan-cancer.

Immunotherapy is the main choice of systemic therapy for many cancer patients. Growing evidence suggests that pyroptosis, necroptosis, and ferroptosis are tightly associated with antitumor immunity^12^. PANoptosis is a coordinated system in which any one pathway can compensate for another depending on the context and the time^37^. Cancer cells undergoing pyroptosis and necroptosis can induce robust anticancer immunity, and the efficacy can be synergized by immune checkpoint inhibitor (ICI) therapy, even in ICI-resistant cancers^12^. Based on the results of previous studies and our research, we next investigated whether PANoptosis scores could predict the response to immunotherapy. The findings showed that PANoptosis score had a better ability to predict immunotherapy response than PD-1, PD-L1, and CTLA-4. In addition, patients in the immunotherapy responder group had significantly higher PANoptosis scores than that of a non-responder group in metastatic melanoma, which was consistent with the result that high scores displayed a protective role in SKCM (Fig. 7). In another two immunotherapy datasets, patients with high PANoptosis scores benefited from the immunotherapy and had better survival outcome. These results suggest that PANoptosis score could be used as a predictive biomarker for immunotherapy response, however, further research is required to verify the prediction value and reveal the potential mechanisms.

In summary, we applied a multi-omics approach to characterize the functional role of PANoptosome complex and its association with the immune landscape in pan-cancer. Our results indicated that PANoptosis genes play a crucial role in tumor progression. They were associated with patients’ survival in a cancer-type-dependent manner. Elevated PANoptosis score was closely correlated with TME and the infiltration level of most immune cells in pan-cancer. In addition, PANoptosis score could act as a biomarker for predicting response to immunotherapy in patients with cancers. The results of the study deepen our understanding of PANoptosis components in cancers and provide insights into the discovery of novel prognostic and immunotherapy response biomarkers.

## Supplementary Information

Supplementary Tables.

## Data Availability

The datasets analyzed during the current study are available in the TCGA database (https://tcgadata.nci.nih.gov/tcga/), GTEx database (https://commonfund.nih.gov/GTEx), TIMER (http://timer.cistrome.org), ImmuCellAI database (http://bioinfo.life.hust.edu.cn/ImmuCellAI), GEO database (https://www.ncbi.nlm.nih.gov), GSCALite (http://bioinfo.life.hust.edu.cn/web/GSCALite/), and MSigDB database (http://software.broadinstitute.org/gsea/msigdb/index.jsp).

## References

[1] Du Toit A (2013). Cell death: Balance through a bivalent regulator. Nat Rev Mol Cell Biol..

[2] Adams JM, Cory S (2007). The Bcl-2 apoptotic switch in cancer development and therapy. Oncogene.

[3] Hanahan D, Weinberg RA (2011). Hallmarks of cancer: The next generation. Cell.

[4] Liu X, Xia S, Zhang Z, Wu H, Lieberman J (2021). Channelling inflammation: Gasdermins in physiology and disease. Nat. Rev. Drug Discov..

[5] Chen X, Li W, Ren J, Huang D, He WT, Song Y (2014). Translocation of mixed lineage kinase domain-like protein to plasma membrane leads to necrotic cell death. Cell Res..

[6] Wang Y, Kanneganti TD (2021). From pyroptosis, apoptosis and necroptosis to PANoptosis: A mechanistic compendium of programmed cell death pathways. Comput. Struct. Biotechnol. J..

[7] Hou J, Zhao R, Xia W, Chang CW, You Y, Hsu JM (2020). PD-L1-mediated gasdermin C expression switches apoptosis to pyroptosis in cancer cells and facilitates tumour necrosis. Nat. Cell Biol..

[8] Newton K, Dugger DL, Wickliffe KE, Kapoor N, de Almagro MC, Vucic D (2014). Activity of protein kinase RIPK3 determines whether cells die by necroptosis or apoptosis. Science.

[9] Taabazuing CY, Okondo MC, Bachovchin DA (2017). Pyroptosis and apoptosis pathways engage in bidirectional crosstalk in monocytes and macrophages. Cell Chem. Biol..

[10] Samir P, Malireddi RKS, Kanneganti TD (2020). The PANoptosome: a deadly protein complex driving pyroptosis, apoptosis, and necroptosis (PANoptosis). Front. Cell Infect. Microbiol..

[11] Malireddi RKS, Karki R, Sundaram B, Kancharana B, Lee S, Samir P (2021). Inflammatory cell death, PANoptosis, mediated by cytokines in diverse cancer lineages inhibits tumor growth. Immunohorizons.

[12] Tang R, Xu J, Zhang B, Liu J, Liang C, Hua J (2020). Ferroptosis, necroptosis, and pyroptosis in anticancer immunity. J. Hematol. Oncol..

[13] Harbron C, Chang KM, South MC (2007). RefPlus: An R package extending the RMA Algorithm. Bioinformatics.

[14] Ulloa-Montoya F, Louahed J, Dizier B, Gruselle O, Spiessens B, Lehmann FF (2013). Predictive gene signature in MAGE-A3 antigen-specific cancer immunotherapy. J. Clin. Oncol..

[15] Riaz N, Havel JJ, Makarov V, Desrichard A, Urba WJ, Sims JS (2017). Tumor and microenvironment evolution during immunotherapy with nivolumab. Cell.

[16] Kim JY, Choi JK, Jung H (2020). Genome-wide methylation patterns predict clinical benefit of immunotherapy in lung cancer. Clin. Epigenet..

[17] Liu CJ, Hu FF, Xia MX, Han L, Zhang Q, Guo AY (2018). GSCALite: A web server for gene set cancer analysis. Bioinformatics.

[18] Hanzelmann S, Castelo R, Guinney J (2013). GSVA: Gene set variation analysis for microarray and RNA-seq data. BMC Bioinformatics.

[19] Diboun I, Wernisch L, Orengo CA, Koltzenburg M (2006). Microarray analysis after RNA amplification can detect pronounced differences in gene expression using limma. BMC Genomics.

[20] Yoshihara K, Shahmoradgoli M, Martinez E, Vegesna R, Kim H, Torres-Garcia W (2013). Inferring tumour purity and stromal and immune cell admixture from expression data. Nat. Commun..

[21] Zeng D, Li M, Zhou R, Zhang J, Sun H, Shi M (2019). Tumor microenvironment characterization in gastric cancer identifies prognostic and immunotherapeutically relevant gene signatures. Cancer Immunol. Res..

[22] Uhlen M, Fagerberg L, Hallstrom BM, Lindskog C, Oksvold P, Mardinoglu A (2015). Proteomics. Tissue-based map of the human proteome. Science.

[23] Hinshaw DC, Shevde LA (2019). The tumor microenvironment innately modulates cancer progression. Cancer Res..

[24] Malireddi RKS, Kesavardhana S, Kanneganti TD (2019). ZBP1 and TAK1: Master regulators of NLRP3 inflammasome/pyroptosis, apoptosis, and necroptosis (PAN-optosis). Front. Cell Infect. Microbiol..

[25] Malireddi RKS, Tweedell RE, Kanneganti TD (2020). PANoptosis components, regulation, and implications. Aging (Albany NY)..

[26] Medema JP, de Jong J, van Hall T, Melief CJ, Offringa R (1999). Immune escape of tumors in vivo by expression of cellular FLICE-inhibitory protein. J. Exp. Med..

[27] Rooney MS, Shukla SA, Wu CJ, Getz G, Hacohen N (2015). Molecular and genetic properties of tumors associated with local immune cytolytic activity. Cell.

[28] Yu S, Dai J, Ma M, Xu T, Kong Y, Cui C (2019). RBCK1 promotes p53 degradation via ubiquitination in renal cell carcinoma. Cell Death Dis..

[29] Liu ML, Zang F, Zhang SJ (2019). RBCK1 contributes to chemoresistance and stemness in colorectal cancer (CRC). Biomed. Pharmacother..

[30] Donley C, McClelland K, McKeen HD, Nelson L, Yakkundi A, Jithesh PV (2014). Identification of RBCK1 as a novel regulator of FKBPL: Implications for tumor growth and response to tamoxifen. Oncogene.

[31] Guo H, Callaway JB, Ting JP (2015). Inflammasomes: Mechanism of action, role in disease, and therapeutics. Nat. Med..

[32] Gu H, Deng W, Zhang Y, Chang Y, Shelat VG, Tsuchida K (2022). NLRP3 activation in tumor-associated macrophages enhances lung metastasis of pancreatic ductal adenocarcinoma. Transl. Lung Cancer Res..

[33] Bae JY, Lee SW, Shin YH, Lee JH, Jahng JW, Park K (2017). P2X7 receptor and NLRP3 inflammasome activation in head and neck cancer. Oncotarget.

[34] Shiao SL, Chu GC, Chung LW (2016). Regulation of prostate cancer progression by the tumor microenvironment. Cancer Lett..

[35] Gong Z, Zhang J, Guo W (2020). Tumor purity as a prognosis and immunotherapy relevant feature in gastric cancer. Cancer Med..

[36] Thorsson V, Gibbs DL, Brown SD, Wolf D, Bortone DS, Ou-Yang TH (2018). The immune landscape of cancer. Immunity.

[37] Ketelut-Carneiro N, Fitzgerald KA (2022). Apoptosis, pyroptosis, and necroptosis—Oh my! The many ways a cell can die. J. Mol. Biol..

